# Oculoplastics and Pediatric Ophthalmology: An Update

**DOI:** 10.4103/0974-9233.63068

**Published:** 2010

**Authors:** Imtiaz A. Chaudhry

**Affiliations:** Senior Academic Consultant, Acting Chief, Oculoplastic and Orbit Division, King Khaled Eye Specialist Hospital, Riyadh, Saudi Arabia

It is with great excitement and pride that I write this editorial for this theme-based issue of *MEAJO*. It was my privilege to be a part of the editorial team and to invite, evaluate, and edit reviews on some of the hot topics in the field of Oculoplastics and Pediatric Ophthalmology. Such theme-based issues allow the ophthalmic community to access various current treatment options and surgical paradigms in a timely manner that may otherwise take years to publish in a textbook.^1^,^2^

In this issue of *MEAJO*, Dr. Kaynak-Hekimhan^3^ from Istanbul discusses some of the noncosmetic uses of botulinum toxin in the management of variety of ocular conditions affecting patients of all ages. Noncosmetic uses of botulinum toxin include the treatment of hemifacial spasms and blepharospasm, temporary treatment of idiopathic and thyroid dysfunction induced upper eyelid retraction, and correction of lower eyelid spastic entropion. Botulinum toxin injections in the lacrimal gland have been found to be effective in the treatment of hyperlacrimation due to different causes. Furthermore, the desired effect of a temporary induction of ptosis is useful in facial nerve paralysis (FNP). Other uses include treatment of hyperhidrosis, migraine, tension-type headaches, and paralytic spasticity. Dr. Kaynak-Hekimhan^3^ highlights the need to carefully evaluate muscles involved in facial dyskinesias, investigating any accompanying pathologies and seeking appropriate consultations with other specialists before treatment.

Capillary hemangiomas are among the most common eyelid and orbital tumors of childhood for which parents seek consultation from their pediatricians. In this issue, Bang and Sebautr^4^ provide a comprehensive review on the understanding of the natural history of periocular capillary hemangiomas, indications, and various forms of treatment. They point out the importance of discussing the risks and benefits of each therapeutic option with the parents.

Correction of congenital ptosis is one of the most difficult challenges faced by the pediatric ophthalmologists and oculoplastic surgeons. In this issue, Allard and Durairaj,^5^ from Colorado, review the current treatment procedures and the management of congenital ptosis. They divide congenital ptosis either as an isolated finding or as a component of many congenital syndromes. They discuss the spectrum of options available to surgeons as well as the reasons for selecting a particular technique. The review also described the various materials available to use such as preserved fascia lata for frontalis sling and the complications of each procedure.

Pediatric preseptal or orbital cellulitis may develop from either contiguous extension from periorbital structures or from endogenous spread. In this issue, Gonzalez and Durairaj^6^ emphasize the need to distinguish preseptal from orbital cellulitis. Furthermore, they stress the distinction between bacterial orbital cellulitis from other postseptal processes. A review of the imaging techniques for diagnosis and various treatment options available for correcting congenital ptosis are cogently presented.

In a review article from the University of Colorado, Gonzalez and Durairaj^7^ reported a meta-analysis of orbital floor fractures focusing on indications and timing of surgical repair, outcomes, and complications. They note that indications for repair of indirect orbital floor fractures may vary by the specialty attending the patients. They also reported the various forms of orbital floor fractures, timing, indications for repair and complications of surgery.

Facial nerve paralysis may occur from several causes that include trauma, infections, or neoplasms. Bell's palsy, the most common cause of facial paralysis, is a diagnosis of exclusion. Alsuhaibani^8^ provides an excellent comprehensive review of FNP, and its management pointing out that patients with such condition may require a multidisciplinary approach for proper patient evaluation and management. He emphasizes that it is crucial to recognize and treat the potentially life-threatening underlying causes.

Retinopathy of prematurity (ROP) is a leading cause of preventable blindness in infancy. Solarte,^9^ in this issue presents a comprehensive review of plus disease and ROP, outlining our current understanding of the clinical presentation, pathophysiology, usefulness of the diagnostic tools, and treatment options in the management of children with aggressive forms of ROP disease. He also notes that the understanding of the vascular changes in patients with ROP may help in designing new treatment strategies, which may be useful in salvaging many of the eyes with severe ROP. The author stresses the need of a standardized, quantitative classification system for vascular changes in the posterior pole in ROP to identify posterior pole changes and provides guidance in early treatment planning.

The absence of an eye in early childhood may have many ramifications including monocular status, aesthetic loss of an eye, and psychosocial challenges for a growing child. Absence of an eye in this age group is also unique in that such loss may result in the absence of stimulus volume necessary for expansion of the orbit, which is essential for the development of facial symmetry. Bernardino,^10^ in his comprehensive review of the problem of anophthalmia, describes both medical and surgical methods to stimulate orbital volume development. Bernardino notes that when choosing a modality to treat an ophthalmic socket, one must consider the status of the affected eye, the contralateral eye, the cause of the loss of the eye, the severity of volume deficit, the age of the child, and psychosocial issues including the support system of the child. Early intervention can lead to very satisfactory results allowing for normal anatomic and psychosocial development.

I was really honored to review these articles and to write this editorial on this theme-based issue of *MEAJO*. In this issue, experts from different continents in the area of pediatric ophthalmology and oculoplastic surgery have undertaken an important service for *MEAJO* readers by providing current updates for which the editorial team is very gracious. The editorial board hopes that the added benefit of delivering a perspective from different regions of the world allows ophthalmologists to taper, alter, or consider alternative treatment paradigms as required. It is believed that their efforts will help in enhancing knowledge for those on the frontline of care of the various conditions presented here.
